# Autonomous intranasal delivery systems for central nervous system therapeutics

**DOI:** 10.1038/s12276-026-01781-5

**Published:** 2026-08-03

**Authors:** Haosheng Shen, Santosh Kumar Srivastava, Nikhil Aggarwal, Matthew Wook Chang

**Affiliations:** 1 NUS Synthetic Biology for Clinical and Technological Innovation (SynCTI), National University of Singapore, Singapore, Singapore; 2 Synthetic Biology Translational Research Programme, Yong Loo Lin School of Medicine, National University of Singapore, Singapore, Singapore; 3 National Centre for Engineering Biology (NCEB), Singapore, Singapore; 4 Department of Microbiology, College of Life Sciences, Nankai University, Tianjin, China; 5 Department of Biochemistry, Yong Loo Lin School of Medicine, National University of Singapore, Singapore, Singapore

**Keywords:** Therapeutics, Neurological disorders

## Abstract

Intranasal delivery provides a rapid, non-invasive route to the central nervous system, bypassing the blood–brain barrier and first-pass metabolism. However, its therapeutic potential remains constrained by the nasal cavity’s complex anatomy, the restricted surface area and permeability of the olfactory epithelium, and short drug residence times. Recent advances in nanotechnology and synthetic biology have enabled the development of autonomous and programmable delivery systems that can target the olfactory epithelium, enhance brain entry and sustain therapeutic release. This review highlights current strategies for engineering intranasal drug delivery vectors that can replicate or extend cellular functions to enable autonomous nose-to-brain drug delivery. These vectors include: synthetic nanoparticles that mimic essential cellular activities and allow for modular surface modification; extracellular vesicles that naturally carry therapeutic cargo and exhibit parent-cell-derived tropism; and living therapeutics, such as engineered microbes, viruses or stem cells, that respond dynamically to host environments and can be genetically programmed for precise payload production. Emphasis is placed on the modular design of functional components, host-responsive interactions tailored to anatomical and physiological cues, and the integration of programmable functions that collectively drive delivery autonomy and therapeutic efficacy. Together, these advances position intranasal delivery as a versatile platform for treating neurological disorders, offering a foundation for future translational development.

## Innovative Approaches to Nose-to-Brain Drug Delivery

Intranasal drug delivery offers a promising route to bypass the blood–brain barrier (BBB) and deliver therapeutics directly to the brain. This review examines emerging intranasal delivery systems that enhance transport, targeting and interaction with brain tissues. It highlights how engineered vectors can improve drug retention in the nasal cavity and support more efficient brain entry, particularly by engaging direct nose-to-brain pathways. Beyond conventional materials, the authors emphasize biologically inspired approaches such as extracellular vesicles, which exploit natural targeting mechanisms, and engineered living systems — including microbes, viruses, and stem cells — that can enable more controlled and responsive drug release. Overall, the review shows how advances in biologically informed design and synthetic biology could expand treatment options for neurological disorders, and improve therapeutic outcomes in challenging brain diseases.

This summary was initially drafted using artificial intelligence, then revised and fact-checked by the author.

## Introduction

The blood–brain barrier (BBB) is a major obstacle to central nervous system (CNS) drug delivery, as it restricts the entry of most therapeutics from the circulation into the brain^1^. To overcome this challenge, intranasal delivery has emerged as a promising non-invasive strategy that enables direct transport of therapeutics to the brain, effectively bypassing the BBB^2,3^. Within the nasal cavity, olfactory sensory neurons connect the olfactory epithelium (OE) to the olfactory bulb of the brain, with their axons traversing the cribriform plate, forming the olfactory pathway for direct nose-to-brain delivery^4–6^. Additionally, the trigeminal nerves, which innervate the respiratory epithelium (RE), provide an additional route for intranasally administered molecules to reach deeper regions of the brain, including the brainstem^6^. Together, these nose-to-brain pathways hold considerable potential for delivering therapeutics to the brain and offer new treatment avenues for a wide range of neurological disorders, including neurodegenerative diseases, psychiatric conditions, brain tumours, stroke and metabolic disorders with neurological involvement.

Despite the advantages of intranasal delivery, nose-to-brain transport remains limited by anatomical complexities and suboptimal delivery efficiency. In particular, the trigeminal pathway poses challenges owing to the diffused and intricate distribution of its nerve endings within the RE and its considerably longer transit time, which complicate the development of precise and effective delivery strategies^7,8^. Additionally, the RE exhibits lower permeability than the OE^9^ and is primarily adapted for mucociliary clearance^10,11^, which reduces the residence time of therapeutics in the nasal cavity, decreasing their uptake. In contrast, the olfactory pathway is considered the primary route for nose-to-brain delivery owing to the well-characterized location and relatively high permeability of the OE. However, in humans, the OE comprises only about 3% of the total nasal cavity surface area and accommodates a limited volume of 25–200 μl^12–14^. Although the total volume of the nasal cavities is approximately 6 cm^3^, the small surface area and restricted access to the OE significantly constrain the amount of drug that can be delivered through this route. Consequently, the majority of intranasally administered molecules are deposited in the RE, where they are either absorbed systemically or eliminated via mucociliary clearance and enzymatic degradation^15,16^.

To overcome the anatomical and physiological barriers inherent to nose-to-brain delivery, a diverse array of intranasal drug-delivery systems have been developed to enhance therapeutic efficacy. Given the restricted surface area and volume of the OE, along with the structural complexity of the nasal cavity, effective delivery systems must combine high drug-loading capacity with autonomous targeting capabilities specific to the OE. Interestingly, the olfactory pathway permits the penetration of nanosized particles typically less than 200 nm in diameter^17^ enabling efficient transport across the nasal mucosa. Nanoparticle encapsulation protects therapeutic molecules from enzymatic degradation, prolongs mucosal residence time and improves overall bioavailability. Recent advances have focused on the integration of biocompatible materials into nanoparticles to enhance mucosal adhesion, facilitate tight-junction penetration and improve OE-targeting specificity. More sophisticated delivery strategies involve the use of extracellular vesicles derived from human cells, which naturally possess cell-specific tropism and intrinsic biological signals that support targeted and efficient delivery. Additionally, emerging approaches utilizing engineered living therapeutics offer the potential for autonomous, sustained and responsive delivery to the brain via the nasal route. However, as delivery systems become more complex and autonomous, they often encounter trade-offs, including reduced drug payload capacity, challenges in scalable manufacturing and increased regulatory hurdles.

This review highlights current strategies employed in intranasal drug-delivery systems aimed at enabling efficient and autonomous nose-to-brain transport of therapeutics. The focus is placed on engineered delivery vectors that actively regulate transport, targeting and tissue interaction. Accordingly, direct intranasal administration of soluble or standalone biologics (e.g. recombinant antibodies or other macromolecules) is not covered. Furthermore, the review focuses on delivery mechanisms rather than on pharmacological or therapeutic suitability of specific cargos. Instead, therapeutic agents are discussed only as functional readouts to illustrate delivery behaviour. Particular emphasis is placed on the foundational design principles that underpin these systems, including the modular integration of functional biomimetic components such as delivery vehicles, targeting ligands and mechanisms for OE penetration. We also examine vector–host interactions shaped by niche- and pathway-specific cues, as well as the tunability of therapeutic payloads through chemical or genetic engineering. Together, these elements contribute to delivery specificity, functional autonomy and therapeutic efficacy. Collectively, these advances offer a comprehensive framework for understanding the evolving landscape of intranasal delivery technologies and underscore their potential in addressing therapeutic challenges within the CNS.

## Synthetic nanoparticles

**Fig. 1:**
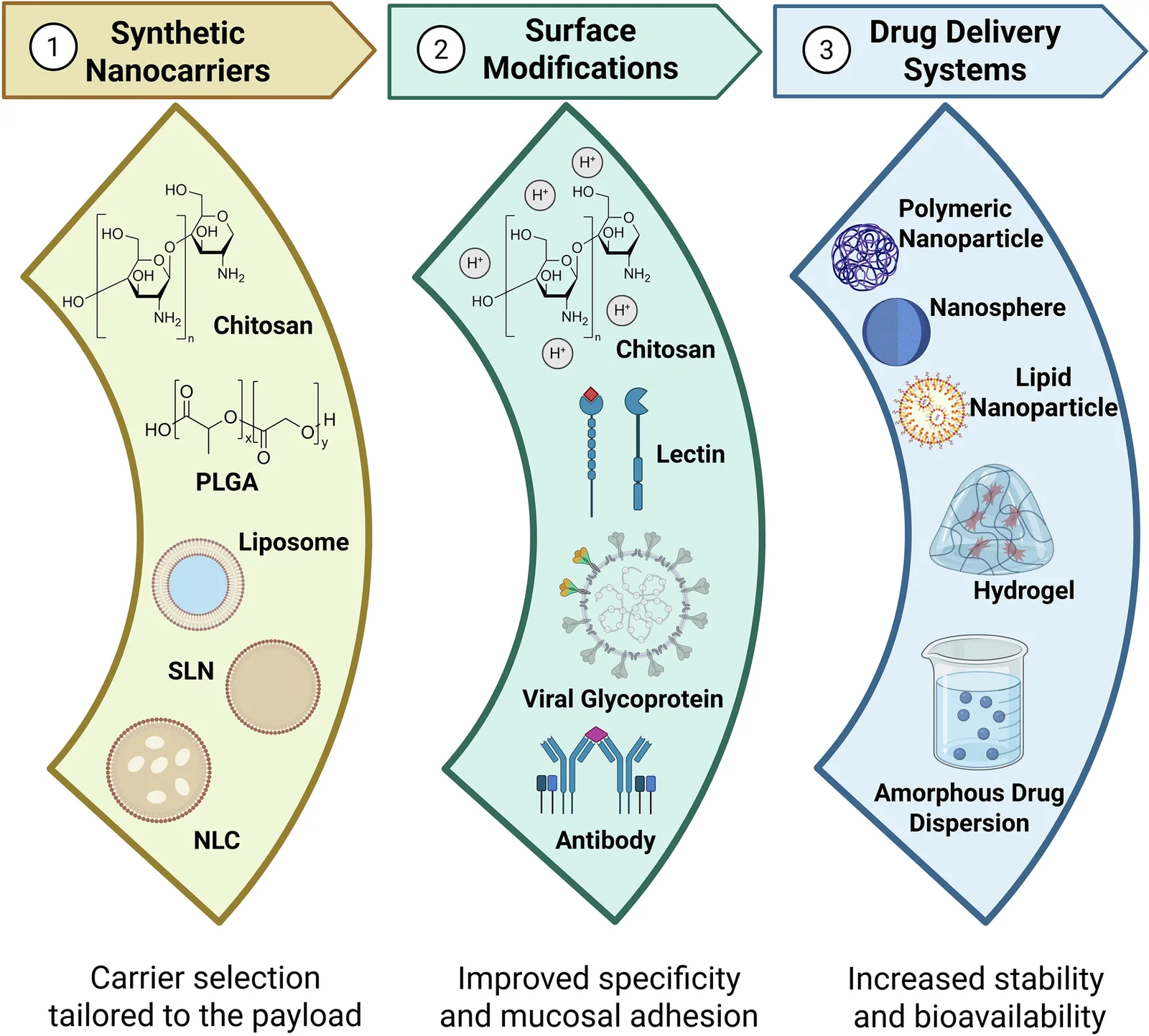
Modular design of synthetic nanoparticles. Synthetic nanoparticles are constructed through the optimization of three key components: the core carrier, the surface modifications and the delivery module. NLC, nanostructured lipid carrier; PLGA, poly (lactic-co-glycolic acid); SLN, solid lipid nanoparticle.

Synthetic nanoparticles represent the most extensively studied and applied class of drug-delivery systems for intranasal administration. The versatility of nanotechnology enables the modular design of these nanoparticles to match specific payload properties, incorporate surface modifications for targeted delivery and achieve optimized formulations for enhanced stability and bioavailability (Fig. 1). The incorporation of functional biomimetic elements enables synthetic nanoparticles to resemble the structural features of living cells, emulating diverse yet relatively basic cellular functions. These modifications allow the nanoparticles to actively engage with host tissues and achieve a limited degree of drug-delivery autonomy (Table 1).

**Table 1:** Comparative overview of intranasal delivery vectors.

Delivery vector	Key features	Advantages	Limitations	Applications	Refs.
Polymeric nanoparticles (e.g. PLGA, chitosan)	Biodegradable polymers; tunable size; mucoadhesive coatings (e.g. chitosan)	Prolonged nasal retention, controlled drug release, scalable manufacturing	Limited delivery autonomy; potential cytotoxicity at high doses	PD, ischaemic stroke	^24–34^
Lipid nanoparticles (e.g. liposomes/SLNs/NLCs)	Lipid-based carriers encapsulating hydrophilic and hydrophobic drugs	High biocompatibility; enhanced CNS penetration; protection from enzymatic degradation	Limited delivery autonomy; physical instability (liposomes); complex formulation	AD, PD, depression	^33–46^
EVs	Naturally secreted vesicles (from MSCs, macrophages, NSCs); intrinsic cell targeting	Relatively high delivery autonomy; low immunogenicity; neuroprotective signalling	Low yield; poor scalability; difficult to engineer	Neurodegenerative disorders, stroke recovery	^68–75^
Stem cells (e.g. MSCs, NSCs)	Self-renewal capacity; secretion of neurotrophic and immunomodulatory factors	High delivery autonomy; homing to injured brain tissue; regenerative effects	Risk of tumorigenesis; high cost; storage and handling challenges	PD, ischaemic stroke, AD	^76–124^
AAVs	Single-stranded DNA viruses; diverse serotypes with CNS tropism	High delivery autonomy; long-term gene expression; efficient CNS transduction	Immune responses; expensive production; limited payload	Gene therapy for neurodegeneration (AD, PD)	^129–137^
Engineered bacteria (e.g. *Lactococcus*, *Lactobacillus* spp.)	Living vectors engineered to secrete therapeutic peptides	High delivery autonomy; cost-effective production; genetic manipulation for versatile payloads; sustaied in situ drug release	Limited clinical safety data for CNS targeting	Obesity, neuroinflammation	^138,139^

AAV, adeno-associated viruses; AD, Alzheimerʼs disease; CNS, central nervous system; EV, extracellular vesicle, MSC, mesenchymal stem cell; NLC, nanostructured lipid carrier; NSC, neural stem cell; PD, Parkinsonʼs disease; PLGA, poly (lactic-co-glycolic acid; SLN, solid lipid nanoparticle.

This section explores the design of such nanoparticles and their interactions with host cells during nose-to-brain delivery. Particular emphasis is placed on representative nanoparticle platforms that incorporate structural or functional elements inspired by living cells, enhancing tissue engagement and delivery autonomy.

### Polymeric nanoparticles

Mucociliary clearance is one of the primary defence mechanisms of the RE, which lines the majority of the nasal cavity. It involves the coordinated action of mucus-producing goblet cells and motile cilia on epithelial cells to trap and transport inhaled particles, pathogens and excess substances toward the nasopharynx for elimination^10,11^. This continuous clearance process serves to protect the underlying tissues from environmental insults and plays a significant role in limiting the retention time and absorption efficiency of intranasally administered therapeutics^11^.

Polymeric nanoparticles are extensively employed in intranasal drug delivery to enhance mucosal adhesion and prolong the residence time of the therapeutics within the nasal cavity. Among them, chitosan, a cationic polysaccharide derived from the deacetylation of chitin, is one of the most widely studied and utilized polymers for this purpose. Owing to its positive surface charge, chitosan nanoparticles interact electrostatically with the negatively charged nasal epithelium rich in mucins and glycoproteins. This interaction significantly enhances mucosal adhesion, thereby extending the retention time of administered therapeutics, improving their local bioavailability and ultimately increasing delivery efficiency to the brain.

Comparative studies have shown that chitosan solutions and microspheres exhibit significantly prolonged mucosal retention times, approximately three- and eight-fold longer than non-mucoadhesive agents in sheep nasal cavities^18^, and two- and four-fold longer in humans^18,19^. To further enhance drug residence time and controlled release, chitosan nanoparticles are often formulated into hydrogels that combine strong mucoadhesive properties with the ability to gradually release drug-loaded nanoparticles upon intranasal administration. To facilitate ease of delivery, many of these hydrogels allow in situ gelation — transitioning from a liquid spray to a semi-solid gel at physiological temperatures. Majcher et al. developed a sprayable in situ-gelling hydrogel composed of starch nanoparticles and O-carboxymethyl chitosan for the intranasal delivery of the antipsychotic peptide PAOPA^20^. This formulation effectively alleviated schizophrenia-associated behavioural abnormalities in a rat model for at least 72 h^20^. In another study, lecithin–chitosan hybrid nanoparticles were incorporated into a thermosensitive methylcellulose gel for the intranasal delivery of piribedil, a drug used for Parkinson’s disease (PD)^21^. Upon administration, the formulation gelled at body temperature, and pharmacokinetic analysis in rats demonstrated a 6.4-fold increase in brain bioavailability of piribedil compared with its solution form^21^.

Despite these positive effects, chitosan nanoparticles often suffer from structural limitations and poor solubility at physiological pH, resulting in loosely packed matrices with relatively low payload capacity^22^. In contrast, synthetic polymers such as poly (lactic-co-glycolic acid) (PLGA) form nanoparticles with more compact and stable structures, enabling higher drug loading, limited degradation and sustained release^23^. PLGA is a biodegradable copolymer composed of polylactic acid (PLA), which is hydrophobic and degrades slowly, and polyglycolic acid (PGA), which is hydrophilic and degrades more rapidly. The physicochemical properties of PLGA can be tailored by adjusting the monomer ratio, allowing for optimized encapsulation of target payloads^24^.

Although PLGA nanoparticles lack intrinsic mucoadhesive properties, their surfaces can be readily functionalized or coated to enhance mucosal adherence for intranasal delivery. For instance, intranasal delivery of chitosan-coated PLGA nanoparticles loaded with the chemotherapy reagent carmustine were used for glioblastoma treatment in rats, resulting in a 2.7-fold increase in the brain-to-blood ratio and a 5.8-fold increase in carmustine’s half-life in brain compared with its suspension^25^. Other studies have reported the surface decoration of PLGA nanoparticles with plant-based lectins to improve the electrostatic charges of the nanoparticles and enhance their mucosal adhesion in nose-to-brain drug delivery, particularly for the treatment of PD^26^ and ischaemic stroke^27^. In addition to lectins, PLGA nanoparticles have also been functionalized with proteins such as lactoferrin^28,29^ and rabies virus glycoprotein^30^, as well as various antibodies to enable receptor-mediated targeting and enhance particle penetration towards the OE. In addition, surface functionalization with antibodies targeting Ephrin type-A receptor^31,32^, as well as tumour necrosis factor-related apoptosis-inducing ligand (TRAIL)^8^, enhanced the cerebral distribution of PLGA nanoparticles to the disease sites and improved therapeutic outcomes in models of glioblastoma and Alzheimer’s disease (AD). These modifications impart more sophisticated properties to the nanoparticles and improve the autonomy of drug-delivery systems, including enhanced targeting of the OE, improved epithelial penetration and increased cerebral distribution of the PLGA nanoparticles.

### Liposomes and solid lipid nanoparticles

Liposomes are spherical nanoparticles composed of one or more phospholipid bilayers that closely resemble cell membranes. They are capable of encapsulating hydrophilic agents within their aqueous core and hydrophobic compounds within the lipid bilayer, offering versatile loading capacity for a wide range of therapeutics. Owing to their membrane fusion capabilities and efficient intracellular delivery, liposomes are particularly well-suited to the transport of nucleic acid-based drugs. Encapsulation within liposomes protects therapeutic agents from enzymatic degradation in the nasal mucosa, whereas surface functionalization can improve mucosal adhesion, facilitate epithelial penetration and enhance targeting specificity for nose-to-brain delivery. Numerous preclinical studies have demonstrated that intranasally administered liposomal formulations can enhance brain bioavailability, improve therapeutic outcomes and reduce systemic side effects.

Dhaliwal et al. developed cationic liposomes encapsulating mRNA and demonstrated efficient intranasal delivery to the mouse brain, achieving widespread distribution and robust expression of the encoded GFP and luciferase throughout the brain tissues^33^. In a separate study, liposomes loaded with imatinib mesylate were evaluated for the treatment of AD in a rat model^34^. Liposomal encapsulation significantly enhanced brain targeting, resulting in a seven-fold increase in brain deposition (as measured by area under the curve, AUC) compared with intranasal administration of the free drug solution. Furthermore, the liposomal formulation prolonged drug retention in the brain, with a reported time to maximum concentration (Tmax) of 6.6 ± 2.3 h, compared with 2.75 ± 2.21 h for the intranasal solution and 1.33 ± 0.5 h for oral administration^34^. Similar to PLGA nanoparticles, liposomes can also be surface-modified to enhance their performance. Modifications with chitosan^35^, polyethylene glycol^36^ and didecyldimethyl ammonium bromide^37^ have been shown to significantly improve liposomal stability and electrostatic interactions with the mucus, thereby markedly enhancing nose-to-brain delivery efficiency.

Despite their advantages, liposomes are prone to physical instability (e.g. aggregation and fusion) and chemical degradation (e.g. hydrolysis and lipid oxidation), which can cause premature drug leakage, disrupt release kinetics and reduce therapeutic efficacy^38,39^. To overcome these limitations, solid lipid nanoparticles (SLNs) have emerged as an alternative lipid-based delivery system. SLNs contain solid lipid cores stabilized by surfactants, typically composed of triglycerides, partial glycerides, fatty acids, waxes and steroids^40^. The lipid matrix remains solid at both room and physiological temperatures. This structural rigidity enhances physical stability, minimizes premature drug release and provides effective protection for the encapsulated therapeutics^40,41^. SLNs are particularly suited to incorporating lipophilic or moderately amphiphilic compounds, although their capacity to load hydrophilic drugs is typically lower than that of liposomes owing to their dense, hydrophobic lipid matrix, which offers limited aqueous compartments for accommodating water-soluble molecules^40,42^. These nanoparticles primarily enter target cells via endocytosis^40,43,44^. In the context of intranasal delivery, the unique properties of SLNs make them a promising platform for nose-to-brain therapeutic applications^45–48^.

For example, Uppuluri et al. developed palmitic acid-based solid lipid nanoparticles stabilized with polyvinyl alcohol and utilized them for the delivery of piribedil to treat PD in a rat model^48^. Combined with a thermo-responsive methyl cellulose in situ gel, the intranasal administration of the formulation increased cerebral concentration of payload piribedil by 4-fold and reduced plasma peak concentration by 2.3-fold compared with plain intranasal suspension, demonstrating improved olfactory targeting and nose-to-brain uptake^48^. In another study, buspirone-loaded SLNs were formulated using Compritol 888 ATO as the lipid and a surfactant mixture of Tween 80 and Poloxamer^45^. The optimized intranasally delivered SLNs demonstrated a 2.18-fold increase in brain AUC compared with intranasal buspirone solution, along with approximately 3.70-fold improvement in brain distribution of buspirone, demonstrated by a higher brain-to-blood buspirone ratio. These findings underscore the potential of SLNs as an effective platform for intranasal drug delivery, with demonstrated improvements in cerebral drug concentrations and targeting efficiency in preclinical models. However, the highly ordered crystalline structure of SLNs also presents certain drawbacks, including limited drug-loading capacity, unexpected drug expulsion and reduced long-term physical stability^49^.

### Nanostructured lipid carriers

Nanostructured lipid carriers (NLCs) are advanced lipid-based nanocarriers composed of a blend of solid and liquid lipids that form an imperfect crystalline matrix, offering improved drug-loading capacity, controlled release profiles, and enhanced long-term physical stability compared with SLNs^50,51^. The incorporation of liquid lipids disrupts the crystalline order of the solid lipid core, creating structural imperfections that enhance drug-loading capacity and reduce drug expulsion during storage, key limitations associated with SLNs^50,51^. This modification also prevents lipid recrystallization and mitigates premature drug release, contributing to a more stable and sustained release profile^50^. Additionally, NLCs improve the encapsulation of both lipophilic and moderately hydrophilic drugs and offer protection against enzymatic degradation, making them a promising platform for targeted drug delivery and controlled release.

Several preclinical studies have demonstrated that intranasal administration of NLCs enhances brain targeting, prolongs drug retention and improves therapeutic outcomes, particularly for neurological disorders. Shehata et al. developed NLCs composed of Precirol ATO 5, oleic acid, Pluronic F-68 (Plx 188) and Tween 80 to deliver astaxanthin intranasally for AD treatment in rat models^52^. These astaxanthin-loaded NLCs (AST-NLCs) showed good physicochemical stability with amorphous drug dispersion and maintained stability for 6 months at 4–8 ± 2°C. They demonstrated a biphasic and sustained release pattern, with only 17.4% released in the first hour and gradual release over 24 h, in contrast to the intranasal AST solution, which released 81.3% within the first hour and nearly all content within 4 h. In vivo, AST-NLCs significantly reduced oxidative stress, neuroinflammation, amyloidogenesis, and apoptosis, while enhancing cholinergic neurotransmission compared with an AST solution^52^. In another study, NLCs containing Precirol ATO 5, oleic acid and Tween 80 substantially enhanced the brain bioavailability of clozapine in a mouse model of schizophrenia, halving the time to brain uptake and increasing intracerebral drug accumulation by 7-fold compared with an oral clozapine tablet^53^. Additionally, Taha et al. developed cod liver oil-based NLCs for the intranasal delivery of zopiclone in the treatment of insomnia, achieving a three-fold reduction in brain enrichment time^54^. Beyond these, intranasal NLCs have been explored for the treatment of epilepsy^55,56^, depression^57,58^, anxiety^59^, migraine^60,61^, multiple sclerosis^62,63^ and meningitis^64^, demonstrating their broad potential as versatile and effective carriers for CNS drug delivery.

Although SLNs and NLCs have a well-established safety profile and are generally recognized as safe (GRAS), potential concerns may arise from the use of certain surfactants^65^. For instance, a previous study reported severe adverse effects on the nasal mucosa in rats after intranasal administration of anionic and cationic NLCs containing sodium deoxycholate. The treatment caused damage to the epithelial lining and induced acute inflammation, as evidenced by inflammatory-cell infiltration in both types of NLCs^66^. Additionally, skin irritation is a commonly reported side effect following topical and dermal administration of NLC-based drugs^65^. On the other hand, NLCs still face significant challenges, particularly in large-scale production, where optimizing lipid composition and ensuring batch-to-batch consistency remain major hurdles^67^.

## Extracellular vesicles

**Fig. 2:**
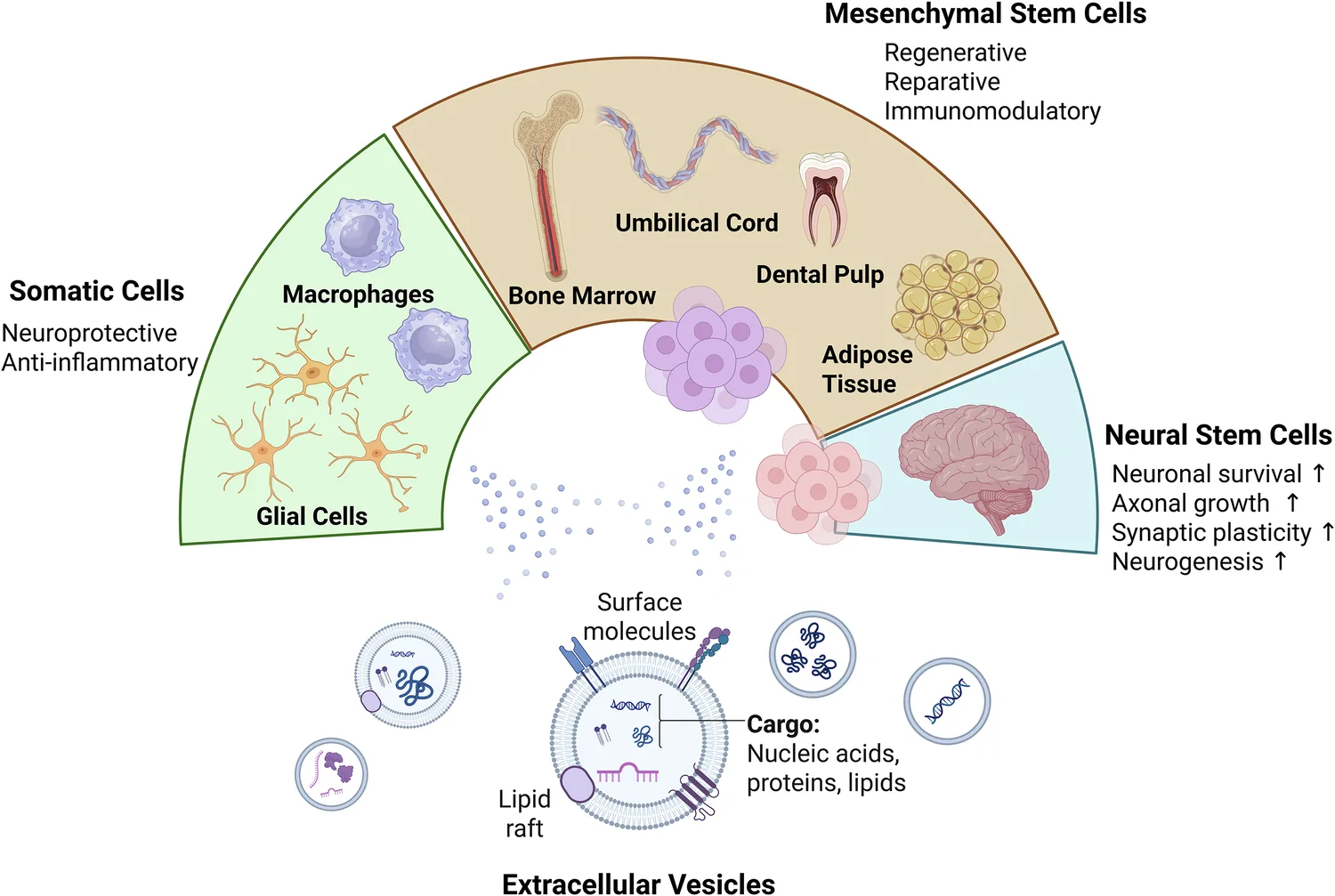
Extracellular vesicles with inherited surface makers and cellular functions. Extracellular vesicles are secreted by somatic cells, mesenchymal stem cells and neural stem cells, each conferring distinct therapeutic properties through their inherited molecular cargo and surface markers.

All living cells secrete extracellular vesicles (EVs) or exosomes as a fundamental means of intercellular communication. These bilayered lipid vesicles carry membrane components and bioactive cargo that closely reflect those of their parental cells, effectively functioning as biomimetic nanoparticles (Fig. 2). Inheriting key traits from their parental cells, EVs possess a relatively high degree of delivery autonomy compared with synthetic nanoparticles and can home to specific niches within the host body (Table 1). Although direct engineering of EVs remains challenging, preconditioning the parental cells can enhance therapeutic efficacy by modulating both the cargo and surface molecules of the released EVs.

Owing to their high biocompatibility and targeting capabilities, EVs have been widely explored for intranasal drug delivery. This section provides an overview of EVs derived from various cell types and examines how their inherited biochemical properties contribute to their roles in facilitating nose-to-brain transport.

### Extracellular vesicles derived from somatic cells

Various somatic cells naturally secrete EVs with preserved biological characteristics and endogenous cargo as their parent cells, making them promising vehicles for therapeutic delivery. Notably, glial-cell-derived EVs, particularly from microglia and astrocytes, play essential roles in CNS communication and exhibit intrinsic neuroprotective and anti-inflammatory properties, especially when secreted in response to pathological conditions such as inflammation. For example, Hu et al. demonstrated that astrocytes exposed to morphine release EVs that impair microglial phagocytosis via Toll-like receptor 7 (TLR7) activation and upregulation of the long intergenic non-coding RNA Cox2 (lincRNA-Cox2)^68^. Intranasal administration of astrocyte-derived EVs loaded with lincRNA-Cox2 siRNA significantly downregulated lincRNA-Cox2 and restored microglial phagocytic activity in morphine-treated mice, highlighting the therapeutic potential of RNA-loaded EVs in substance abuse-related neurodegeneration^68^. In another study, astrocyte-derived EVs stimulated with acidic fibroblast growth factor and administered intranasally conferred neuroprotection in AD animal models by reducing amyloid-β burden, promoting neurite outgrowth and improving cognitive performance^69^. These benefits were mediated through downregulation of miR-206-3p and subsequent upregulation of brain-derived neurotrophic factor (BDNF), which suppressed δ-secretase activity and alleviated AD pathology^69^. Similarly, Arvanitaki et al. reported that in DNA-repair-deficient and aged mice, microglia accumulate cytosolic double-stranded DNA, which is packaged into EVs and transferred to neurons, triggering a neurotoxic immune response^70^. To mitigate this, microglia-derived EVs were engineered to deliver recombinant DNase I via intranasal administration, effectively degrading cytosolic double-stranded DNA, reducing neuroinflammation and neuronal apoptosis, and delaying the onset of neurodegenerative symptoms^70^.

In addition to CNS-resident somatic cells, macrophage-derived EVs are particularly appealing owing to their innate tissue penetration and the scalability of macrophage cultures for large-scale production. These vesicles are enriched with bioactive molecules such as cytokines, microRNAs and enzymes, which may synergize with additional therapeutic payloads to enhance treatment efficacy^71,72^. In one study, biotinylated catalase-loaded macrophage EVs administered intranasally successfully targeted key brain regions, such as the prefrontal cortex, corpus callosum and hippocampus, without inducing histological or macroscopic abnormalities, demonstrating both safety and therapeutic potential in neurodegenerative diseases^73^. In addition, macrophage-derived EVs also benefit from the natural homing capacity of macrophages toward sites of inflammation and injury, enhancing therapies towards neurodegenerative diseases. In a previous study, Haney et al. developed catalase-loaded macrophage EVs and delivered them intranasally in a PD mouse model, resulting in significant reductions in neuroinflammation, improved dopaminergic neuron survival and a marked decrease in motor dysfunction (from 150 to 26 rotations per minute)^74^. In another related study, EVs derived from autologous macrophages engineered to express glial-cell-line-derived neurotrophic factor (GDNF) were administered intranasally and led to enhanced locomotor function, increased neuronal survival and reduced neuroinflammation in a PD mouse model, with no observable off-target toxicity^75^.

Overall, somatic-cell-derived EVs represent a versatile and biologically relevant class of intranasal therapeutics, leveraging their endogenous cargo and innate cell-specific properties to modulate neuroinflammation, support neuronal survival and deliver treatment directly to the brain via intranasal delivery.

### Extracellular vesicles derived from mesenchymal stem cells

Mesenchymal stem cells (MSCs) are widely employed to produce EVs owing to their strong therapeutic potential, which arises from a diverse cargo of bioactive molecules including proteins, lipids and nucleic acids that mediate regenerative, reparative and immunomodulatory effects. MSC-derived EVs can be efficiently isolated from accessible sources such as bone marrow, umbilical cord and dental pulp, and are amenable for large-scale in vitro expansion. To enhance their therapeutic efficacy, MSCs are often preconditioned with disease-mimicking stimuli such as pro-inflammatory cytokines, e.g. TNF-α and IFN-γ, to enrich EVs with specific therapeutic payloads^76,77^. Studies have demonstrated that EVs derived from human bone marrow and umbilical cord MSCs can cross the BBB via intranasal administration, reaching key regions of the CNS, including the hippocampus and cerebral cortex^78^. In AD mouse models, intranasally delivered medium-conditioned MSC-derived EVs reduced amyloid-β plaque burden, preserved spatial learning and memory, and mitigated neuroinflammation^78–80^. Similarly, intranasal administration of these MSC-derived EVs has shown therapeutic benefits in preclinical models of PD^81,82^, autism spectrum disorder^83–85^, ischaemic stroke^86,87^ and CNS injuries^88,89^.

Adipose tissue is another abundant and readily accessible source of MSC-derived EVs. Compared with bone marrow and umbilical-cord blood, adipose tissue contains a higher frequency of MSCs, allowing for more efficient isolation and expansion^90^. Moreover, adipose tissue can be collected via liposuction, which is a minimally invasive and less painful procedure than bone-marrow aspiration, enhancing its clinical applicability^91^. Adipose MSC-derived EVs are well tolerated, exhibit low immunogenicity and are enriched with neuroprotective and anti-inflammatory cargos, including cytokines, growth factors and regulatory microRNAs^92,93^. Rohden et al. demonstrated that intranasally administered EVs derived from human adipose MSCs significantly reduced infarct volume, preserved BBB integrity and promoted neovascularization in a rat model of ischaemic stroke^94^. These EVs distributed widely throughout the brain and led to substantial improvements in behavioural performance, including motor symmetry, memory function and anxiety reduction, suggesting long-lasting neuroprotection^94^. Intranasal delivery of adipose MSC-derived EVs has also been shown to stimulate neuronal growth and improve autism spectrum disorder-like behaviours in mice, effects linked to elevated levels of BDNF and anti-inflammatory signalling^95^. Additionally, adipose MSC-derived EVs have shown therapeutic benefits in models of progressive multiple sclerosis by promoting remyelination through inhibition of the NLRP3–p38/MAPK proinflammatory signalling axis, and in amyotrophic lateral sclerosis by restoring motoneurons and neuromuscular junction integrity through the suppression of apoptotic pathways^96,97^.

Interestingly, the physiological and pathological roles of adipose-derived EVs in the CNS are influenced by variables such as adipose tissue depot, donor health status and EV molecular composition. For example, although EVs derived from healthy adipose tissue exhibit neuroprotective effects, those from obese or metabolically dysregulated individuals may contribute to neuroinflammation, synaptic dysfunction and cognitive decline^98–100^. These dual characteristics underscore the importance of source optimization and molecular profiling in the therapeutic application of adipose MSC-derived EVs for neurological diseases.

### Extracellular vesicles derived from neural stem cells

EVs derived from neural stem cells (NSCs) possess unique properties that render them particularly promising for therapeutic applications targeting the CNS. Owing to their neural lineage, NSC-derived EVs are intrinsically adapted to the CNS microenvironment and carry brain-enriched surface markers and signalling molecules that may enhance cellular uptake by neurons, astrocytes, oligodendrocytes and microglia^101^. The multipotent nature of neural stem cells, which allows them to differentiate into key neural-cell types, is likely to contribute to the enrichment of their EV cargo with bioactive molecules involved in neural development and regeneration. These EVs are rich in neurotrophic factors and regulatory microRNAs that promote neuronal survival, axonal growth, synaptic plasticity and neurogenesis, offering functional advantages over EVs derived from non-neural sources such as MSCs^102–104^. In a comparative study using a murine thromboembolic stroke model, NSC- and MSC-derived EVs, derived from the same pluripotent stem cell line, exhibited similar physical characteristics and systemic biodistribution profiles^105^. However, NSC-derived EVs demonstrated superior therapeutic efficacy, including enhanced modulation of systemic immune responses, greater neuroprotection and improved functional recovery post-stroke^105^. These findings underscore the potential of NSC-derived EVs as a tailored and potent therapeutic platform for CNS repair and regeneration.

Several preclinical studies have demonstrated the therapeutic potential of NSC-derived EVs for treating brain disorders via intranasal administration. Notably, EVs derived from human induced pluripotent stem cell (iPSC)-derived NSCs were detected in virtually all subregions of the forebrain, midbrain and hindbrain within 45 min of intranasal delivery, with preferential internalization into neurons, interneurons and microglia^106^. Another study showed that intranasally delivered iPSC-derived NSC-EVs were taken up by neurons, astrocytes and microglia across all major brain regions in adult rats and mice, and significantly enhanced hippocampal neurogenesis^107^. In a mouse model of diet-induced cognitive dysfunction, NSC-derived EVs restored CREB/BDNF/TrkB signalling in the hippocampus and reversed memory impairment caused by a high-fat diet^108^. Furthermore, Wang et al. engineered NSC-derived EVs to overexpress the chemokine receptor CXCR4 and loaded them with anti-miR-21 and miR-100, achieving targeted delivery to glioblastoma regions enriched in CXCL12 and effectively inhibiting glioblastoma growth^109^. These findings underscore the versatility of NSC-derived EVs as a platform for neuro-regeneration and targeted treatment of CNS pathologies. However, their broader clinical application is currently constrained by limited accessibility and scalable production methods, which are largely dependent on iPSC-derived NSC sources. Addressing these challenges will be critical for translating the promise of NSC-EVs into effective and widely available therapies for neurological diseases.

## Live therapeutics

**Fig. 3:**
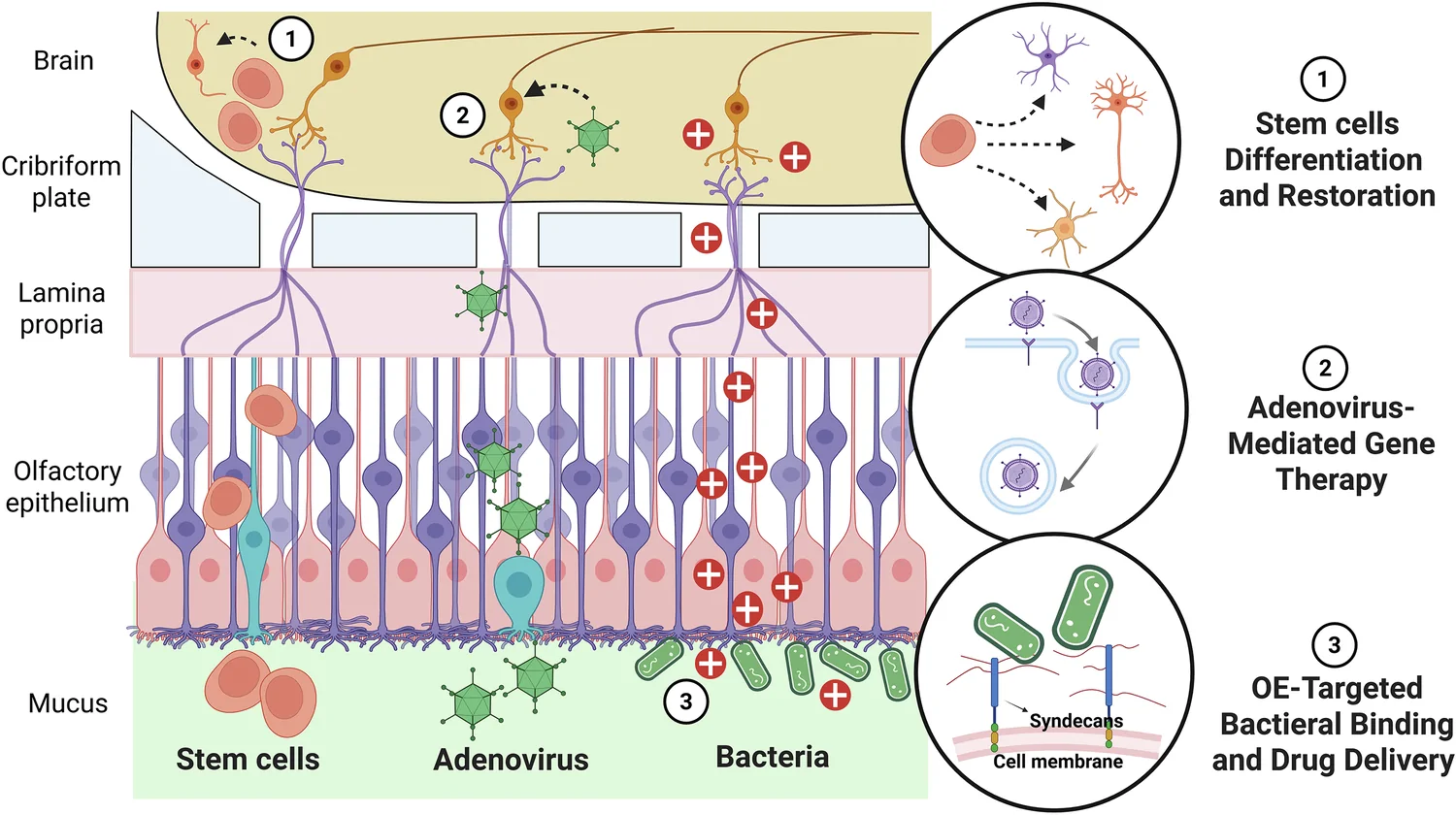
Niche- and pathway-specific nose-to-brain drug delivery by live therapeutics. Stem cells, viruses and bacteria used as living therapeutics exhibit distinct central nervous system recognition patterns and employ different mechanisms of therapeutic delivery. OE, olfactory epithelium.

Living therapeutics have recently emerged as an innovative strategy for intranasal drug delivery. The nasal cavity hosts a complex microenvironment comprising various host cells and a diverse community of commensal microorganisms. Leveraging this biological niche, recent studies have explored the use of live agents, including stem cells, viruses and bacteria, as intranasal delivery vehicles for nose-to-brain transport. Compared with other delivery vectors, these organisms exhibit high levels of autonomy (Table 1), navigating and functioning according to niche- and pathway-specific cues, which enables organism-specific design of autonomous drug-delivery systems (Fig. 3). In addition, their compatibility with stable genetic engineering enhances their versatility, allowing for the targeted delivery of a broad spectrum of therapeutics.

This section reviews existing research on such systems, with a focus on the mechanisms by which they mediate therapeutic efficacy, targeting specificity, and sustained delivery. The potential of living therapeutics to expand the capabilities of intranasal drug-delivery platforms is also discussed in the context of future clinical applications.

### Stem cells

Stem cells have emerged as transformative therapeutic tools in regenerative medicine and are applied via the intranasal route owing to their intrinsic capabilities for self-renewal, differentiation into neural lineages and the secretion of bioactive factors that support repair and neuroprotection. In contrast to their EVs, which are on the nanoscale and diffuse across nasal barriers, stem cells are substantially larger and access the brain through active migration along the olfactory and trigeminal nerves into the brain^110^.

As discussed earlier, MSCs are among the most accessible stem-cell types, readily harvested from bone marrow, umbilical cord, adipose tissue and dental pulp. Owing to their low immunogenicity, robust paracrine signalling and secretion of neurotrophic factors, MSCs have become the most extensively studied cell type for intranasal administration in CNS therapies^111,112^. Preclinical studies have demonstrated that intranasally delivered MSCs can migrate to multiple brain regions, including the olfactory bulb, hippocampus and cortex, where they contribute to improved neurological outcomes in models of PD, ischaemic stroke and AD^112–114^. In PD models, dopaminergic neurons in the substantia nigra degenerate, leading to reduced tyrosine hydroxylase (TH) expression. TH is the rate-limiting enzyme in dopamine biosynthesis, catalysing the conversion of tyrosine to L-DOPA, which is converted to dopamine. Intranasally delivered MSCs in PD models have been shown to migrate into the substantia nigra, restore TH expression and improve motor performance^114^. Similarly, Wei et al. demonstrated that bone-marrow-derived mesenchymal stem cells (MSCs) rapidly migrated to infarcted brain regions within 1.5 h following intranasal administration. The observed therapeutic benefits were primarily attributed to efficient brain targeting, paracrine secretion of neurotrophic and angiogenic factors, and modulation of endogenous repair mechanisms, collectively leading to a significant reduction in infarct volume, enhanced angiogenesis and improved neurological outcomes in an ischaemic stroke model^115^. In AD models, both MSCs and their secreted EVs localized to amyloid-β plaques in the hippocampus and cortex, modulated neuroinflammation and improved memory performance^80^. Notably, a comparative study by Ohtaki et al. reported that intranasally administered MSCs produced neuroprotective effects comparable with those of intracranial injection, while offering a safer, non-invasive alternative^116^. These findings were further supported by subsequent studies showing reduced microglial activation and enhanced neuronal regeneration in models of neonatal hypoxic–ischaemic encephalopathy. Beyond cell replacement and neuroprotection, intranasal delivery of MSCs is also recognized for its potent anti-inflammatory effects owing to the release of the anti-inflammatory mediators, having been shown to downregulate pro-inflammatory cytokines such as IL-1β, IL-6, TNF, and IFNγ in neurodegenerative models, thereby reducing glial activation and enhancing neuroprotection^112,117^.

NSCs are also under active investigation for their therapeutic potential in intranasal delivery owing to their inherent neurogenic potential. NSCs can differentiate into neurons, astrocytes and oligodendrocytes, making them suitable for treating neurodegenerative conditions such as PD, glioblastoma and brain injury^118,119^. Reitz et al. and Dey et al. demonstrated that intranasally administered NSCs can reach glioblastoma tissue within 6 to 24 h and persist in the tumour microenvironment for several days^119,120^. Importantly, these NSCs can be genetically engineered to express therapeutic payloads such as pro-apoptotic ligands, cytokines such as interferon-beta, or oncolytic viruses, which are then selectively delivered to tumour cells while minimizing systemic toxicity^121^. The combination of intranasal delivery and engineered NSCs thus represents a minimally invasive, repeatable strategy for targeted treatment of glioblastoma and other intracranial malignancies^122^. In addition, pluripotent stem cells, such as iPSCs reprogrammed from somatic cells, offer considerable promise owing to their ability to differentiate into all neural lineages and their potential for autologous, immune-compatible therapy^123^.

Despite numerous advantages, stem-cell-based therapies have limitations, including infection, graft rejection and the formation of teratomas^124–127^. Therefore, further safety and efficacy studies are essential before their translation via intranasal delivery.

### Adeno-associated viruses

Adeno-associated viruses (AAVs) are powerful gene therapy vectors capable of eliminating or replacing pathogenic genes and enabling sustained expression of therapeutic transgenes through efficient cellular transduction. These small, single-stranded DNA viruses comprise at least 13 known serotypes, each with distinct tissue tropisms. Traditionally, CNS applications of AAVs required invasive delivery methods such as direct intracerebral injection. Although viral particles are nanosized and are known to enter the CNS via the olfactory pathway^128,129^, AAVs have been actively explored for intranasal delivery to achieve targeted and non-invasive gene transfer to the brain^130^. AAV transport efficiency is governed by capsid interactions with specific receptors expressed on nasal epithelial and olfactory pathway cells. Distinct AAV serotypes bind heparan sulfate proteoglycans, sialylated glycans, galactose residues and the universal AAV receptor (AAVR/KIAA0319L), triggering receptor-mediated endocytosis. High-affinity receptor engagement enhances mucosal uptake and promotes vector trafficking along olfactory- and trigeminal-nerve pathways, enabling efficient nose-to-brain delivery^8,131,132^. Importantly, AAV-mediated drug delivery to the brain following intranasal administration does not result in uniform CNS distribution but instead exhibits pronounced region-specific and serotype-dependent biodistribution^133^. Initial vector uptake occurs at the OE, where AAVs enter olfactory sensory neurons and supporting cells, followed by axonal transport to the olfactory bulb, which serves as a principal gateway to the CNS^134^. Subsequent dissemination to distal brain regions is mediated through multiple, partially overlapping mechanisms, including olfactory- and trigeminal-nerve pathways, cerebrospinal fluid circulation, and anterograde and retrograde neuronal transport^135^. Biodistribution is strongly influenced by capsid–receptor interactions, serotype-specific neuronal tropism and regional cellular permissiveness. Consequently, serotypes with high affinity for neuronal receptors and efficient axonal transport, such as AAV9, achieve broader CNS dissemination, whereas others, including AAV2, exhibit more localized transduction near olfactory regions. Additional regional differences in receptor expression, extracellular matrix composition and cellular uptake capacity further shape transgene distribution and expression levels, critically determining therapeutic efficacy in nose-to-brain AAV gene and drug-delivery applications^135,136^. Several serotypes with strong CNS tropism, including AAV2, AAV8 and AAV9, have demonstrated successful intranasal delivery, achieving efficient transduction of target brain cells and therapeutic benefits in preclinical models of neurological diseases.

Among all serotypes, AAV2 has a broad tropism and transduces a wide range of cell types and tissues, owing to its high affinity to its widespread primary receptors, heparan sulfate proteoglycans. Several preclinical studies have demonstrated the intranasal application of AAV2 vectors for gene therapy targeting the CNS. Moreover, numerous preclinical and clinical investigations have confirmed its favourable safety profile, characterized by low immunogenicity and minimal toxicity. In AD models, intranasal delivery of AAV2 encoding the neuroprotective peptide CBD3 has been shown to significantly reduce both amyloid-β plaques and tau pathology, the two major hallmarks of AD. This approach presents a promising, non-invasive strategy for halting or potentially reversing neurodegeneration^137^. Furthermore, intranasal delivery of recombinant AAV2 vectors encoding neurotrophic genes such as BDNF and neurotrophin 4 has shown significant antidepressant and neuroprotective effects in rodent models of depression, restoring BDNF expression and promoting hippocampal neurogenesis. Liu et al. reported that AAVs encoding neurotrophin 4 improved behavioural outcomes in socially isolated mice, enhanced synaptic proteins (synaptophysin, PSD-95) and reduced neuroinflammation and oxidative stress^138^.

In addition to AAV2, AAV8 and AAV9 show great tropism towards the CNS and have been extensively studied for intranasal delivery of gene therapy. For instance, in a-L-iduronidase deficient mucopolysaccharidosis type I (MPS I) disease model, intranasal delivery of AAV8 expressing a recombinant iduronidase was able to increase a-L-iduronidase activity in the olfactory bulb of the mice^139^. Another study by Belur et al. demonstrated that intranasal delivery of AAV9 expressing a-L-iduronidase led to increased iduronidase enzyme levels in the brain in MPS I mice, subsequently improving cognitive outcomes. This research is particularly significant as MPS I is a lysosomal storage disorder with severe neurological manifestations, and effective enzyme delivery to the brain has been a major challenge^140^.

Collectively, these studies highlight that intranasal AAV-mediated gene therapy offers a non-invasive and efficient approach to delivering therapeutic genes to the brain, modulating neurotrophic signalling pathways, and alleviating depressive symptoms by enhancing neural plasticity and reducing neuroinflammation. These findings underscore the potential of intranasal AAVs to treat a broad range of neurological and psychiatric disorders by promoting neuronal survival, synaptic remodelling and functional recovery. The success of this approach, however, is influenced by multiple factors including serotype selection, vector dosage and tissue tropism^141^. Optimal serotype selection must align with the target cell population and therapeutic objectives, whereas dosing requires careful calibration to achieve sufficient gene expression without eliciting detrimental immune responses. Ongoing efforts focus on engineering AAV vectors with enhanced CNS tropism and reduced immunogenicity, alongside the development of refined dosing strategies to improve safety and therapeutic efficacy.

### Bacteria

The use of engineered bacteria as living therapeutics represents an innovative and rapidly evolving strategy in biomedical research. The nasal cavity harbours one of the densest and most diverse microbiomes in the respiratory tract, comprising both commensal and potentially pathogenic microorganisms. Although the direct entry of bacteria into the CNS poses safety risks, some nasal commensals are uniquely positioned at the OE, where they can interact with host cells with frequent exchange of metabolites. These metabolites may traverse the OE and access the CNS, offering a non-invasive route for drug delivery. Although still an emerging area, the application of engineered bacteria for direct CNS targeting via the intranasal route holds significant promise, particularly for sustained, localized production and delivery of therapeutic agents.

The safety of intranasally administered probiotic species has been extensively studied, with a primary focus for respiratory vaccination^142^. Beyond immunization, engineered *Lactococcus lactis* strains have been applied intranasally to deliver recombinant leptin for obesity management in murine models^143^. Engineered leptin-producing *L. lactis* was capable of modulating antigen-specific immune responses and regulating body weight and food intake in vivo. More recently, our group demonstrated the utility of the human commensal *Lactobacillus plantarum* WCFS1 (Lp) as a living vehicle for nose-to-brain drug delivery^144^. Following intranasal administration, Lp was shown to selectively localize to the OE via specific binding to N-acetyl heparan sulfate expressed on the apical surface of the OE. Lp strains were engineered to release therapeutic payloads directly within the OE, enabling subsequent accumulation in the lamina propria and translocation to the olfactory bulb. In mice maintained under a high-fat diet, Lp strains expressing appetite-regulating peptides, including leptin, ɑ-melanocyte-stimulating hormone and BDNF, led to reduced food intake, attenuated weight gain and improved glucose homeostasis.

This strategy combines the natural nasal epithelial tropism of commensal bacteria with precise genetic engineering, thereby expanding the therapeutic landscape for neurodegenerative, psychiatric and metabolic CNS disorders. Beyond the direct recombinant expression and secretion of protein-based therapeutics, advances in synthetic biology have enabled bacterial vectors to adopt versatile drug-delivery modalities. These include surface display for the attachment and controlled release of complex chemical compounds^145–147^, as well as the production of bacterial membrane vesicles for the delivery of nucleic acids^148–151^. Although these advanced modalities have yet to be tested in the context of intranasal delivery, they offer significant potential for developing next-generation, non-invasive CNS therapies.

#### Clinical translation challenges and limitations of intranasal central nervous system therapeutics

Despite encouraging preclinical outcomes, the clinical translation of intranasal CNS therapeutics remains constrained by multiple physiological, anatomical and technological barriers. The nasal cavity constitutes a highly dynamic environment characterized by rapid mucociliary clearance, enzymatic degradation and limited dosing volume, all of which restrict drug residence time and compromise delivery reproducibility^152,153^. Although advanced delivery vectors can improve mucosal adhesion, stability and epithelial penetration, low and variable bioavailability persists, as substantial fractions of administered drugs are lost through swallowing, mucociliary transport or systemic absorption^153,154^.

Anatomical differences further complicate clinical translation^13^. The composition of olfactory and respiratory mucosa differs markedly between rodents and humans, with the olfactory region comprising approximately 40–50% of the nasal surface area in rodents but only about 3–10% in humans^155,156^. These anatomical disparities significantly influence drug deposition and transport dynamics, limiting the predictive value of small-animal studies and necessitating validation in large-animal models^152^. Even when deposition is achieved, the proportion of therapeutics that reach the brain via olfactory or trigeminal pathways remains difficult to quantify, and off-target distribution to the respiratory tract or systemic circulation poses safety concerns^8^.

Moreover, entry into nose-to-brain pathways does not guarantee delivery to the intended neural targets. Transport involves multiple interconnected processes, including epithelial uptake, intracellular trafficking, axonal transport and redistribution through cerebrospinal fluid and extracellular spaces^7,157^. Each step imposes distinct constraints on spatial distribution, often resulting in heterogeneous brain exposure with preferential accumulation near entry regions. Although engineered vectors may enhance specific transport steps, predictable and uniform CNS distribution remains challenging, particularly for indications requiring engagement of deep or widespread neural networks.

Biodistribution is further shaped by cargo-specific properties such as molecular size, charge, stability and receptor affinity. These features influence uptake, retention and clearance, leading to substantial variability even within the same delivery platform^8,153,158^. For example, proteins and neurotrophins may exhibit rapid degradation, receptor-mediated sequestration or dose-limiting peripheral effects, whereas nucleic-acid cargos may be constrained by endosomal escape, intracellular trafficking and cell-type-dependent uptake leading to distinct spatial and cellular patterns of delivery, even when carried by the same platform^7,159,160^. Accordingly, the field would benefit from moving beyond “brain delivery” as a binary endpoint towards quantitative, region-resolved pharmacokinetics and pharmacodynamics, using standardized imaging, tissue mapping and functional target engagement readouts that enable comparison across studies^7,8^. These issues are particularly consequential for AAV-mediated gene therapies, where therapeutic success depends on serotype/capsid tropism, receptor availability and neuronal permissiveness, all of which shape a non-uniform, region-dependent pattern of transduction following intranasal administration^130,132^.

Modality-specific limitations also introduce additional barriers. EVs and nanomaterials often suffer from heterogeneous cargo loading, unclear long-term biodistribution and challenges in scalable manufacturing under regulatory standards^51,161^. Viral vectors are constrained by immunogenicity and neuroinflammatory risks, whereas cell-based approaches raise concerns regarding persistence, immune activation and fate control^162–164^. Although advances in vector engineering continue to alleviate some of these constraints, fundamental challenges related to dose control, safety, reproducibility and large-scale production remain unresolved.

Collectively, these considerations indicate that although delivery vectors can partially mitigate physiological and anatomical barriers, intranasal CNS translation requires coordinated optimization of pathway entry, intra-brain distribution, and long-term safety. Addressing these interconnected challenges will be essential to realizing the full therapeutic potential of intranasal-delivery platforms.

## Conclusion

Intranasal delivery represents a compelling non-invasive strategy for accessing the CNS by partially bypassing the BBB and avoiding first-pass metabolism. Yet, its clinical impact remains constrained by physiological barriers, including limited olfactory surface area, rapid mucociliary clearance, anatomical variability and inconsistent regional targeting. Recent advances in synthetic biology and nanotechnology have enabled autonomous, programmable delivery platforms ranging from nanostructured materials and EVs to viral and living therapeutics that expand the possibilities for CNS-targeted interventions. However, translation will require more than technological innovation. Achieving reproducible dosing, precise regional delivery, long-term safety, and scalable manufacturing remain essential for clinical feasibility. Moving forward, integrating mechanistic insight with clinically driven design through coordinated optimization of vector, cargo and administration strategies will be key to transforming intranasal delivery from experimental promise into a practical therapeutic modality for neurological disease.
